# Laparoscopic duodenojejunostomy requiring a side-to-side jejunojejunostomy in malignant stenosis of the gastrojejunal anastomosis in jejunal cancer: A case report

**DOI:** 10.1016/j.ijscr.2020.08.002

**Published:** 2020-08-19

**Authors:** Yugo Matsui, Teppei Murakami, Satoshi Ishida, Ryuichi Mikami, Shotaro Matsuda, Aoi Tayama, Ryutaro Sakata, Takehisa Harada, Masahiko Takeo

**Affiliations:** Department of Surgery, Kobe City Medical Center West Hospital, 2-4 Ichibancho, Nagata-ku, Kobe, 653-0013, Japan

**Keywords:** Laparoscopic duodenojejunostomy, Malignant stenosis, Jejunojejunostomy, Jejunal cancer, Opioid-induced bowel dysfunction, Case report

## Abstract

**Introduction:**

Laparoscopic duodenojejunostomy is a common surgical treatment for SMA syndrome. Although there are successful cases of laparoscopic duodenojejunostomies on malignant conditions, cancer patients with opioid-induced bowel dysfunction could struggle in maintaining an oral diet despite surgical treatment of the mechanical obstruction.

**Case presentation:**

A 66 year-old woman with a chemotherapy history of 18 months for Stage 4 jejunal cancer near the ligament of Treiz presented with vomiting and dehydration. She had a gastrojejunostomy constructed prior to the induction of chemotherapy. CT scan and endoscopic studies confirmed the stricture of this anastomosis due to tumor invasion. Laparoscopic duodenojejunostomy was performed, but tolerable food intake was not achieved, likely due to limited bowel movements caused by opioid use and tumor invasion of the celiac plexus. A side-to-side jejunojejunostomy was constructed, since accumulation of food in her jejunal loop was thought to be a significant cause of her limited food intake and vomiting. She was able to tolerate oral intake after the second intervention and was discharged home.

**Discussion:**

Successful cases of laparoscopic duodenojejunostomy in malignant strictures of the duodenum have been reported. In this case, the outcome was not so well due to limited bowel movements caused by opioid use. Literature review of laparoscopic duodenojejunostomy on SMA syndrome revealed some cases to be unsuccessful in enabling oral feeding or resolving nausea, and methods to treat such cases could be discussed further.

**Conclusion:**

Laparoscopic duodenojejunostomy is an option for malignant strictures of the duodenum, but a favorable outcome could not be achieved in our case. A side to side anastomosis of the jejunal loop and the efferent jejunum may help in improving the outcome.

## Highlights

• Laparoscopic duodenojejunostomy is a common intervention for the stricture of the distal duodenum.
• Not all patients respond to this intervention, and methods to treat them must be discussed.
• Opioid-induced bowel dysfunction caused fluids to accumulate in the jejunal loop in our case.
• Jejunojejunostomy was a solution to treat our patient.

## 1 Introduction

Laparoscopic duodenojejunostomy has been reported as a surgical intervention for strictures of the 3rd portion of the duodenum, mostly caused by superior mesenteric artery syndrome [1] but also malignant conditions such as locally recurring pancreatic cancer [2]. This method has been shown to be effective in the treatment of SMA syndrome, but there are also reports on poor results at 6-month follow-up [8]. In addition, patients treated with opioids for cancer-related pain can have opioid-induced bowel dysfunction with limited gut motility and segmentation [3], which may interfere with oral intake despite surgical treatment of the mechanical obstruction. Hence, methods to treat unsuccessful cases should be discussed further. We hereby report an unsuccessful case of laparoscopic duodenojejunostomy on a patient with malignant stricture of the gastrojejunal anastomosis, which required additional treatment with a side-to-side jejunojejunostomy. This work has been reported in line with the SCARE criteria [4].

## 2 Case presentation

**Fig. 1:**
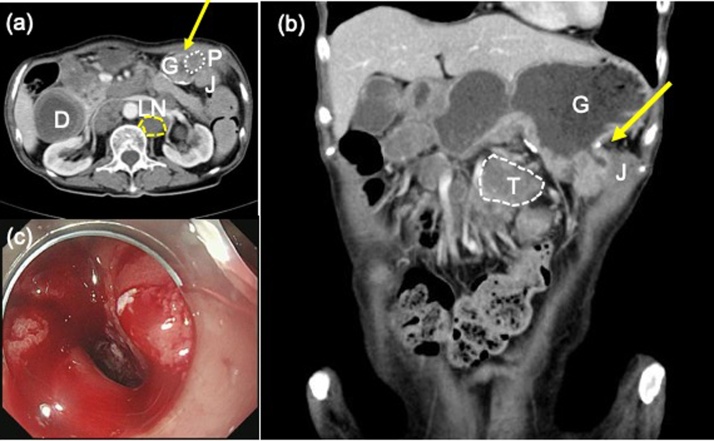
Pre-operative CT, endoscopic findings. (a, b) CT scan reveals stricture of the gastrojejunal anastomosis (yellow arrow) due to peritoneal dissemination (P/white dotted line). The stomach (G) and duodenum (D) are dilated. The primary tumor (T/white dotted line) and lymph node metastasis (LN/yellow dotted line) is also seen. J: jejunum. (c) Endoscopy revealing invasion of the tumor into the lumen of the anastomosis.

The patient is a 66 year-old woman with jejunal cancer near the ligament of Treitz diagnosed 20 months ago. The initial evaluation revealed metastasis in her para-aortic as well as suprapancreatic lymph node, thus diagnosed as stage 4, and 1st line therapy was chemotherapy. Due to tumor obstruction, a gastrojejunostomy was constructed prior to chemotherapy. FOLFOX + Bevacizumab was started 18 months ago. The regimen was changed 4 months ago to FOLFIRI + Ramucirumab due to disease progression. There was also a complaint of left epigastric pain 7 months ago suspected to be cancer-related, for which treatment with opioids was started. In the course of her treatment, she presented with vomiting and dehydration. Contrast-enhanced computed tomography (CT) revealed a dilated gastroduodenum and a collapse of the efferent jejunum loop, indicating obstruction at the gastrojejunostomy. This was thought to be caused by a disseminated tumor invading into the anastomosis (Fig. 1a, b). She was hospitalized on the same day. Gastrointestinal endoscopy revealed an ulcer formation at the site of the anastomosis, which suggested tumor invasion into the lumen (Fig. 1c). Adenocarcinoma cells were detected in the biopsy of this ulcer. Stent placement was thought to pose a risk of perforation, and surgical intervention was considered.

**Fig. 2:**
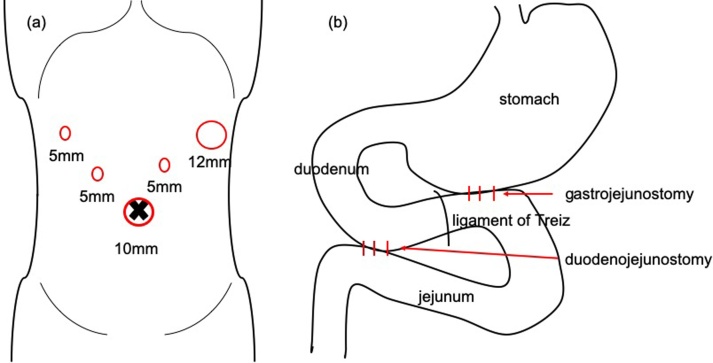
Schematic illustration of (a) port placement and (b) anatomy of anastomosis in laparoscopic duodenojejunostomy.

**Fig. 3:**
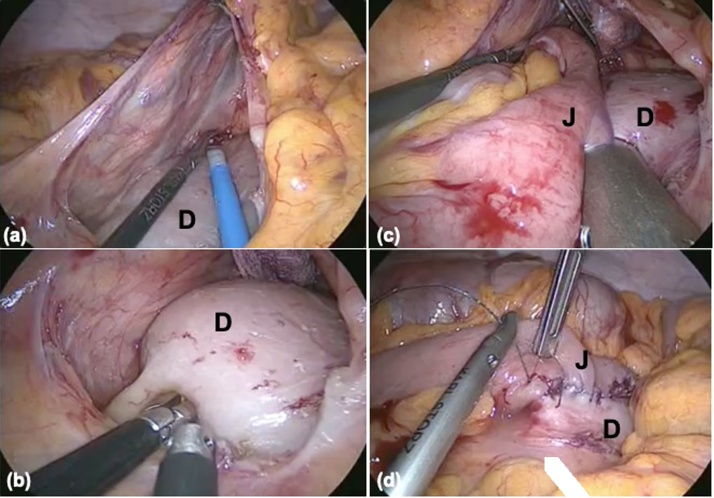
Laparoscopic images of duodenojejunostomy. (a) The transverse colon is retracted cranially, and the second to third portion of the duodenum (D) is exposed by excision of the mesentery. (b) Enterotomy is created in the duodenum and the jejunum. (c) Side-to-side anastomosis is formed with 45-mm stapling device. (d) The enterotomy is closed using a 3-0 Vloc™ suture.

Laparoscopic duodenojejunostomy was performed a week after admission. Decompression with a nasogastric tube was done for 3 days prior to surgery. A 5-port procedure (Fig. 2a) was taken with the operator on the left side of the patient. A 10 mm port was used for the operator’s right hand. Laparoscopic findings revealed no apparent peritoneal dissemination, and the jejunum anal to the gastrojejunal anastomosis was easily mobilized. The transverse colon was retracted cranially, and the third portion of the duodenum was visualized through the colonic mesentery. We divided the overlying peritoneum and the duodenum was separated from the colonic mesentery along the second and third portion. We chose to anastomose the jejunum about 30 cm anal to the gastojejunal anastomosis. The jejunum was brought to the exposed duodenum and anastomosed side-to-side (Fig. 2b), with a stapling device (Signia™ with 45 mm purple reload). The enterotomy was closed with a continuous absorbable V-Loc™ suture (Fig. 3). No abdominal drains were placed, and the nasogastric tube was not removed. Operation time was 151 min, with trivial bleeding.

**Fig. 4:**
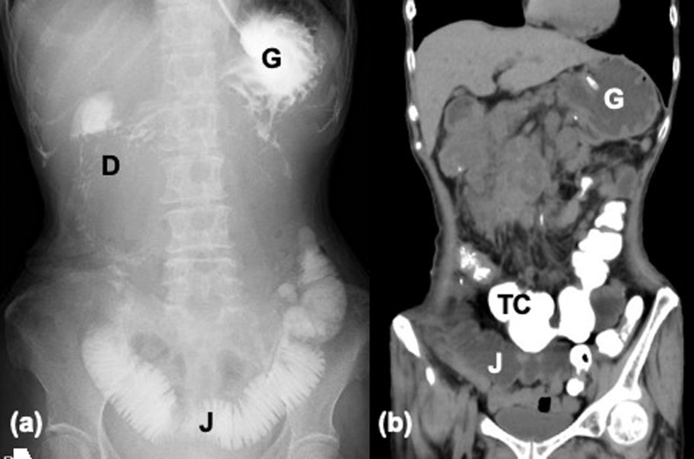
Radiological images after duodenojejunostomy. (a) Gastrografin accumulated in the stomach (G), duodenum (D) and afferent jejunal loop (J). (b) CT scan on the following day showed accumulation of dye in the transverse colon (TC).

Anastomotic passage could not be confirmed in an assessment with gastrografin on post-op day 3, but since no significant reflux was observed for 2 days, oral feeding was started. Repeated vomiting occurred 5 days later and a nasogastric tube was placed (Fig. 4). With the results seen in Fig. 4, bowel dysmotility was suspected, which was thought to be caused by stasis from opioid treatment.

**Fig. 5:**
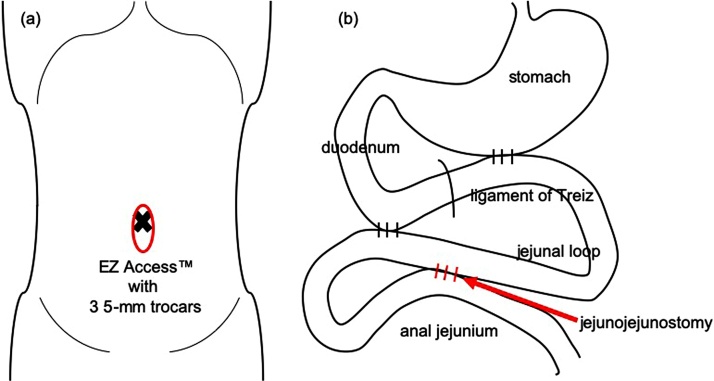
Schematic illustration of (a) port placement and (b) anatomy of anastomosis in laparoscopic side-to-side jejunojejunostomy.

**Fig. 6:**
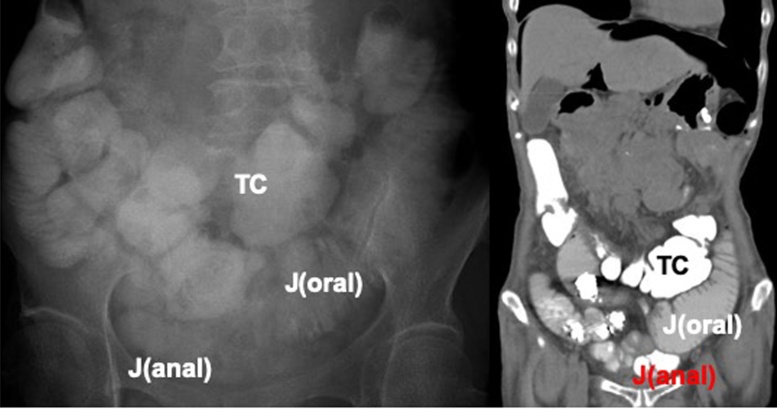
Radiological images after jejunojejunostomy (a) Gastrograffin passing from the jejunal loop (J(oral)) into the jejunum anal to the anastomosis (J(anal)). Contrast material given 2 days prior was seen in the transverse colon (TC). (b) CT scan taken 4 h after gastrograffin injestion.

Surgical intervention was thought to be essential for symptom improvement, and thus laparoscopic side-to-side jejunostomy of the jejunal loop and the jejunum anal to the duodenojejunal anastomosis was performed 30 days after the previous surgery. The EZ Access™ system was inserted into the umbilicus via the open method, and 3 trocars were used (Fig. 5a). The dilated jejunal and the jejunum anal to the duodenojejunostomy was brought to the wound site. A side-to-side anastomosis (Fig. 5b) was done outside the abdomen using a linear stapler (Echelon™ with 60 mm white reload for enterotomy, 60 mm blue reload for defect closure). Operation time was 66 min with trivial bleeding. Radiological studies were conducted on POD 2, but gastric motility was insufficient for a decent assessment. Re-examination on POD 4 revealed contrast material flowing into the anastomosed jejunum, and most of the accumulation was seen in the colon on a CT scan 4 h later (Fig. 6). Meal consumption started on POD 10, and although intake was limited, there was no vomiting. The patient was discharged home 39 days after the second surgery with a follow-up by a home care physician. She required total parenteral nutrition (TPN), but was able to eat until her death 8 days after discharge.

## 3 Discussion

Anastomotic strictures pose a threat to surgeons in a variety of surgical methods, including gastrectomy, colorectal resection, etc. In laparoscopic Roux-en-Y gastric bypass for obesity, rates of anastomotic strictures occur in 5–15% [5,6]. When faced with an anastomotic stricture, physicians are posed with the option of stent placement or surgical intervention. In terms of stent placement versus gastrojejunostomy in malignant gastric outlet obstruction, anastomosis is the favorable choice for patients with a prolonged survival [7]. A multicenter randomized trial SUSTENT revealed that endoscopic stents are mostly indicated for patients with poor performance status, high surgical risk, and life expectancy less than 2 months [14]. Cusati et al. reported a case series of gastrojejunal anastomotic strictures treated by stent placement where all patients ultimately underwent surgical intervention [15]. In our institution, surgery is chosen in most cases of malignant gastric outlet obstruction. Exceptions are if the patient has a short prognosis (less than 30 days), is incapable of tolerating surgery under general anesthesia, e.g. ASA-PS (American Society of Anesthesiologists physical status) score of 4 or greater, or if informed consent could not be obtained. Our patient was expected to continue chemotherapy with a prognosis of 12 months, with ASA-PS 2, and so it is reasonable to say that surgery was the choice of treatment.

Laparoscopic duodenojejunostomy is a surgical method well reported for SMA syndrome. Single-center case series show no mortality, no anastomotic leaks, and a mean post-operative length of stay ranging from 4.5 to 7.5 days [1,[9], [10], [11], [12], [13]] (Table 1). There are also literature reports on laparoscopic duodenojejunostomy performed on malignant duodenal strictures, in which food intake was achieved within a week [2,8]. In our case, oral feeding seemed to have been achieved within a week, but ended in cyclic vomiting, which was thought to be due to limited bowel movement from opioid use. In Chang et al.’s study of SMA syndrome, symptom relief could not be achieved in 12/18 cases in their intermediate follow-ups, for which they argue that pre-operative work up of the other underlying conditions is important in evaluating whether a patient will respond to duodenojejunostomy [9]. In addition, 18% of the patients who received laparoscopic duodenojejunostomy from the studies in Table 1 had nausea or vomiting of various degrees in their post-operative follow-up. In our case, the bowel flow was partially regained by a side-to-side jejunojejunostomy, indicating that the rate-limiting step in bowel flow was in the jejunal loop. As in our case, oral intake free of parenteral or tube feeding may be a difficult goal to achieve in patients with functional dysmotility. The initial goal of our patient was to enable oral feeding and continue chemotherapy, but since this seemed impossible, we hoped to reduce the frequency of nausea and allow supportive care at home. Although hospital stay was much longer than expected, our patient was able to spend her last days at home without nausea. In this sense, the jejunojejunostomy was a solution to her disposition.

**Table 1:** Post-operative complications and persistence of vomiting/nausea in recent case-series studies.

Source	Number of Patients (LDJ performed)	Mean length of stay (days)	Anastomotic leak	Mortality	Complication (number of cases)	Vomiting/Nausea in postoperative follow-up (vomit/nausea)
Kirby et al. [1]	4 (3)	7.5	none	none	gastroparesis (1)	1/0
					ulcer bleeding at anastomosis (1)	
Valiathan et al. [11]	6 (3)	N.A.	none	none	none	2/0
Chang et al. [9]	18 (16)	6.7	none	none	prolonged ileus (2)	1/4
					intolerance to oral intake (2)	
					closed loop obstruction (1)	
Jain et al. [10]	22 (22)	7.27	none	none	delayed gastric emptying (4)	0/0
					ileus (1)	
Pator Peinado et al. [12]	4 (4)	4.5	none	none	melena with mild anemia (1)	1/0
Kim et al. [13]	2 (2)	5.5	none	none	none	0/0

LDJ: laparoscopic duodenojejunostomy.

Although laparoscopic duodenojejunostomy was not successful in achieving the initial goal of our patient, there are reported cases in which symptom improvement or resolution and oral nutrition was gained in patients with SMA syndrome and malignant strictures, suggesting that it is the choice of operation. However, methods to treat cases in which symptoms persist should be discussed further.

## 4 Conclusion

Laparoscopic duodenojejunostomy is a common surgical intervention of SMA syndrome, but there are also cases which failed to enable oral feeding or resolve nausea. Appreciable food intake and bowel flow may not be achieved in patients with limited bowel movements, and methods to treat unsuccessful cases should be discussed further. In our case, a side-to-side jejunojejunal anastomosis involving the loop was a solution.

## Funding

None.

## Ethical approval

Non required.

## Consent

Written informed consent was obtained from the patient for publication of this case report and accompanying images. A copy of the written consent is available for review by the Editor-in-Chief of this journal on request.

## Author contribution

Yugo Matsui: Responsible for literature review, writing and manuscript preparation. Operator of both surgeries.

Teppei Murakami: Responsible for manuscript preparation. Assisted in the laparosope-assisted jejunojejunostomy.

Satoshi Ishida, Ryuichi Mikami: Assisted in the laparoscopic duodenojejunostomy.

Shotaro Matsuda, Aoi Tayama, Ryutaro Sakata, Takehisa Harada, Masahiko Takeo: Responsible for manuscript review.

## Registration of research studies

Not applicable.

## Guarantor

Yugo Matsui.

## Provenance and peer review

Not commissioned, externally peer-reviewed.

## Declaration of Competing Interest

The authors report no declarations of interest.
